# Active Electromagnetically Induced Transparency Effect in Graphene-Dielectric Hybrid Metamaterial and Its High-Performance Sensor Application

**DOI:** 10.3390/nano11082032

**Published:** 2021-08-10

**Authors:** Fan Gao, Peicheng Yuan, Shaojun Gao, Juan Deng, Zhiyu Sun, Guoli Jin, Guanglu Zeng, Bo Yan

**Affiliations:** 1 Collaborative Innovation Center for Bio-Med Physics Information Technology of ZJUT, Zhejiang University of Technology, Hangzhou 310023, China; gaofan@zjut.edu.cn (F.G.); 2111909015@zjut.edu.cn (P.Y.); gsjzjut@163.com (S.G.); jdeng@zjut.edu.cn (J.D.); 2112009041@zjut.edu.cn (Z.S.); j1025264409@163.com (G.J.); 2112009103@zjut.edu.cn (G.Z.); 2 Department of Physics, Zhejiang University of Technology, Hangzhou 310023, China

**Keywords:** electromagnetically induced transparency, graphene-dielectric hybrid metamaterial, refractive index sensing

## Abstract

Electromagnetically induced transparency (EIT) based on dielectric metamaterials has attracted attentions in recent years because of its functional manipulation of electromagnetic waves and high refractive index sensitivity, such as high transmission, sharp phase change, and large group delay, etc. In this paper, an active controlled EIT effect based on a graphene-dielectric hybrid metamaterial is proposed in the near infrared region. By changing the Fermi level of the top-covered graphene, a dynamic EIT effect with a high quality factor (Q-factor) is realized, which exhibits a tunable, slow, light performance with a maximum group index of 2500. Another intriguing characteristic of the EIT effect is its high refractive index sensitivity. In the graphene-covered metamaterial, the refractive index sensitivity is simulated as high as 411 nm/RIU and the figure-of-merit (FOM) is up to 159, which outperforms the metastructure without graphene. Therefore, the proposed graphene-covered dielectric metamaterial presents an active EIT effect in the near infrared region, which highlights its great application potential in deep optical switching, tunable slow light devices, and sensitive refractive index sensors, etc.

## 1. Introduction

Electromagnetically induced transparency (EIT) is the quantum interference effect firstly observed in atomic systems, which weakens the light absorption at the atomic resonance frequency and introduces a narrow transmission window in the broad absorption spectrum [1]. Although firstly discovered in the quantum optics region, this concept was later extended to optical resonant systems, such as photonic crystals [2], whispering-gallery-mode resonators [3], and metamaterials [4]. Stringent experiment conditions like ultracold temperatures and stable coherent lights are not necessary in the resonant systems, which makes them perfect platforms to research the EIT-like effect. Functional manipulations of electromagnetic waves can be easily realized with unique performance like strong dispersion and a large group delay, which has great potential for applications in slow light devices [5,6], nonlinear optics [7], optical sensing [8,9], and optical storage [10,11]. However, metallic metamaterials usually find it difficult to achieve high transmission, Q-factor, and group index in Terahertz and near-infrared regions due to the ohmic loss of metals as well as the radiative loss of surface modes [12]. In this condition, high-refractive-index dielectric materials stand out with low non-radiation loss, which provides a better solution towards high-performance EIT metamaterials. For example, Si resonators are used to obtain a strong EIT-like effect with almost complete transmittance and a high Q-factor of up to 483 [13]. In the dielectric metamaterial, light interacts with high-permittivity Si to generate Mie resonance rather than dipole or LC resonances in metallic components, therefore an extraordinary electromagnetic response without metallic loss can be attained, which guarantees its high Q performance [14].

In recent years, the active tuning and manipulation of electromagnetic waves has been research highlights in the field of metamaterials. By using active materials like phase-change materials [15] and photosensitive semiconductors [16], a tunable EIT-like effect with demanded functions can be easily achieved. Among all the functional materials, two-dimensional (2D) materials, represented by graphene, stand out with dynamic optical and electrical properties [17,18,19]. The Fermi level of graphene can be easily tuned by electrical gating or chemical doping methods, and its high carrier mobility and tunable conductivity are especially beneficial in active optical devices. Up until now, graphene has been used as a tunable component in metal-dielectric metamaterials [20,21,22]. Recently, in the all-dielectric metamaterial region, using graphene to actively control the EIT effect has been studied in the terahertz band [23,24] and the near-infrared band [25,26]. In addition, due to the narrow transmission peak produced by the EIT effect, the peak position shifts sensitively with the slight changing of the environmental refractive index. Therefore, the EIT effect is of great significance in the high-performance sensor application [13], which needs to be further explored.

In this work, we propose a graphene-dielectric hybrid metamaterial with active modulation of the EIT effect. The metamaterial presents a sharp EIT transmission peak with a high Q-factor value in the near-infrared region, and the transmission window can be dynamically tuned by changing the Fermi level of graphene. An excellent slow light effect is attained in the graphene-covered metamaterial with a maximum group delay and group index of 1.6 ps and 2500, respectively. Meanwhile, the high-performance refractive index sensing characteristic is also observed, of which the sensitivity and FOM can reach up to 411 nm/RIU and 159, respectively. By comparing the metastructures with and without graphene, it is found that the top-covered graphene can help improve the sensing performance due to the graphene plasmonics excited in the near infrared. Therefore, the proposed hybrid metamaterial exhibits great application potential in optical switches, slow-light devices, and refractive index sensors, etc.

## 2. Method

**Figure 1:**
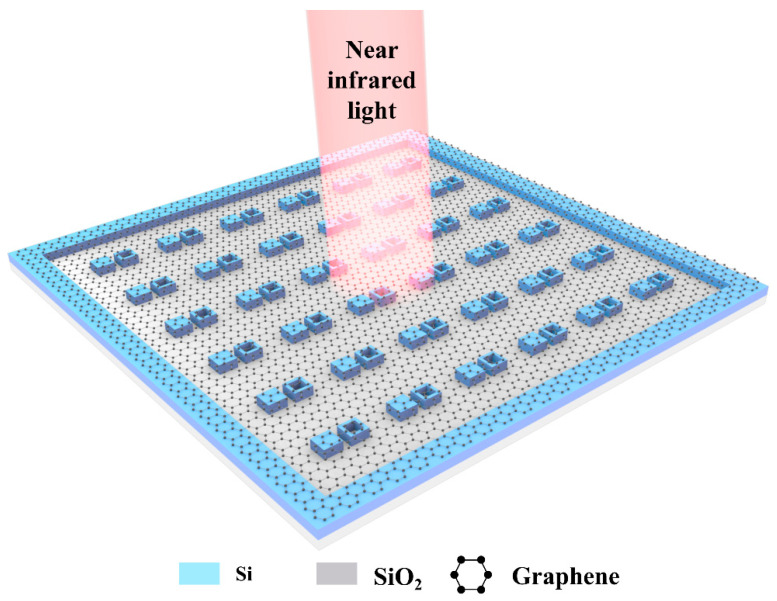
The schematic illustration of the proposed metamaterial, which consists of a solid nanocube (SNC), a hollow nanocube (HNC), and a monolayer graphene and quartz substrate.

The schematic diagram of the designed all-dielectric metamaterial is shown in Figure 1. The metamaterial unit cell consists of a solid nanocube (SNC) and a hollow nanocube (HNC) made from Si, which are placed on the quartz substrate and are covered by monolayer graphene. All-dielectric nanostructures are widely used because they are easily fabricated by using a top-down method [13]. Graphene layers grown from chemical vapor deposition (CVD) can be transferred to dielectric metamaterials using standard transfer techniques [27]. In order to make graphene flat on top of the metamaterial, a Si frame is designed on the periphery with the same height of the unit cells to support the monolayer graphene, as shown in Figure 1. The simulation software CST Microwave Studio is applied to simulate the proposed metamaterial by using a finite element frequency domain solver. Periodic boundary conditions are adopted in the x and y directions, and the z direction is set as the open boundary for light incident and emission. A y-polarized plane wave is applied to illuminate the metamaterial along the -z direction. Besides, the refractive index of Si is set as $n_{Si}=3.7$ [28] and the refractive index of the quartz substrate is set as $n_{SiO_{2}}=1.45$ [29].

**Figure 2:**
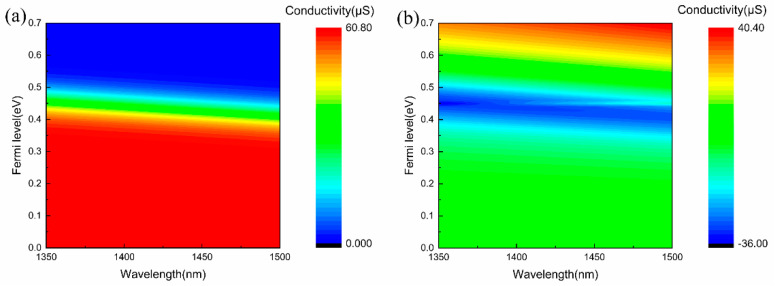
The relationship of graphene’s conductivity with different incident wavelengths and Fermi levels. (**a**) Real part of the conductivity. (**b**) Imaginary part of the conductivity.

For graphene, both intra-band and inter-band transitions contribute to its complex surface conductivity, which can be calculated by the theory of random phase approximation in the local limit and is described as following [30]:

(1) $$\sigma_{g}\left(\omega \right)=\frac{2e^{2}k_{B}T}{\pi \hbar^{2}}\frac{i}{\omega +i\tau^{-1}}\ln\left[2\cosh\left(\frac{E_{F}}{2k_{B}T}\right)\right]+\frac{e^{2}}{4\hbar }\left[\frac{1}{2}+\frac{1}{\pi }\arctan\left(\frac{\hbar \omega -2E_{F}}{2k_{B}T}\right)-\frac{i}{2\pi }ln\frac{{\left(\hbar \omega +E_{F}\right)}^{2}}{{\left(\hbar \omega -E_{F}\right)}^{2}+{\left(2k_{B}T\right)}^{2}}\right]$$

where *e* is the electron charge, $k_{B}$ is the Boltzmann constant, T is the temperature, ℏ is the reduced Plank’s constant, ω is the frequency of the incident light, τ is the relaxation time, and $E_{F}$ is the Fermi level of the graphene. In this case, *T* is assumed to be 300 K, $E_{F}$ is set from 0 eV to 0.6 eV, and $\tau =\left(\mu E_{F}/e\nu_{F}^{2}\right)$ is calculated from Fermi velocity $\nu_{F}=1\times 10^{6}\;m/s$ and the carrier mobility $\mu =10,000\;{\mathrm{cm}}^{2}/\left(V\cdot s\right)$ [30]. Firstly, we discuss the conductive properties of the graphene for a better understanding of its electromagnetic behaviors. The real and imaginary parts of graphene’s conductivity is calculated with different incident wavelengths and Fermi levels, as shown in Figure 2. As we can see from the graphs, when the Fermi level is less than half of the photon energy at the Dirac point ($E_{F}$ < *ℏω*/2), the incident photon is absorbed by graphene due to the inter-band absorption, resulting in a large real part of the graphene conductivity. On the contrary, when the Fermi level is greater than half of the photon energy ($E_{F}$ > *ℏω*/2), the contribution of the inter-band transition is prevented due to the Pauli exclusion principle. Therefore, once the Fermi level exceeds the critical value, the real part of the graphene conductivity will decrease sharply and the imaginary part will continue to increase, resulting from the intra-band transition, as shown in Figure 2b. Due to the intrinsic nature of graphene, it will bend between the resonators, but within the research scope of this paper, the bending of graphene has almost no effect on its dielectric properties and EIT performance [31].

## 3. Results and Discussion

### 3.1. EIT Effect of All-Dielectric Metamaterial without Graphene

**Figure 3:**
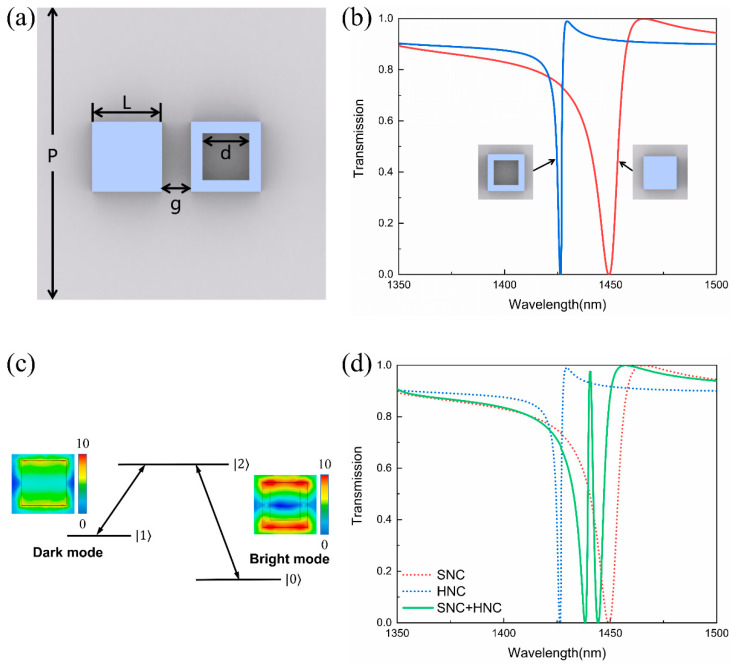
(**a**) Geometric parameters of the dielectric metamaterial without graphene: *P* = 1500 nm, L = 364 nm, d = 244 nm, g = 150 nm. (**b**) The simulated transmission spectra of the separate SNC and HNC. (**c**) Schematic of interference between the bright- and dark-mode resonators. Inset: Electric field distribution of the bright and dark modes at resonant wavelength. (**d**) The simulated transmission spectra of the proposed metamaterial.

Figure 3a shows that the geometric parameters of the unit cell are *P* = 1500 nm, L = 364 nm, d = 244 nm, g = 150 nm, and the heights of the Si nanocubes and quartz substrate are both 190 nm. Firstly, we studied the optical properties of the SNC and HNC separately. Figure 3b is the transmission spectrum of the SNC and HNC under y-polarized light incidence. Here we can find that both SNC and HNC can be excited by the incident light, but the excitation of the SNC is weaker than the HNC due to its broader line width. Similarly, from the electric field distribution in the inset of Figure 3c, it can be seen that the electric field of SNC is weaker than that of HNC. Therefore, SNC can be defined as the dark mode and HNC as bright mode. The interference between the bright and dark modes forms a typical three-level resonant system, as shown in Figure 3c. Here, |0〉, |1〉, and |2〉 represents the ground state, metastable state, and excited state in the three-level system, respectively. In our case, the bright mode can be directly excited (path: |0〉→|2〉), and the excitation of the bright mode can be coupled to the dark mode, resulting in the indirect excitation of the dark mode (path: |0〉→|2〉→|1〉→|2〉). The two modes will destructively interfere under certain conditions, causing a narrow EIT-like transmission peak to appear at the original transmission dip. With the combination of SNC and HNC in one unit cell, the bright and dark modes couple to generate a sharp transmission peak at 1440.4 nm in the Fano resonance dip, as shown in Figure 3d, which is ascribed to the typical EIT phenomenon. Further calculation reveals the transmission amplitude of the EIT peak is as high as 97.5% and the Q-factor is up to 646. Here, the Q-factor is calculated by $Q=\lambda_{0}/FWHM$, where $\lambda_{0}$ is the wavelength of the EIT window and FWHM is the full width at half maximum of the EIT transmission peak.

**Figure 4:**
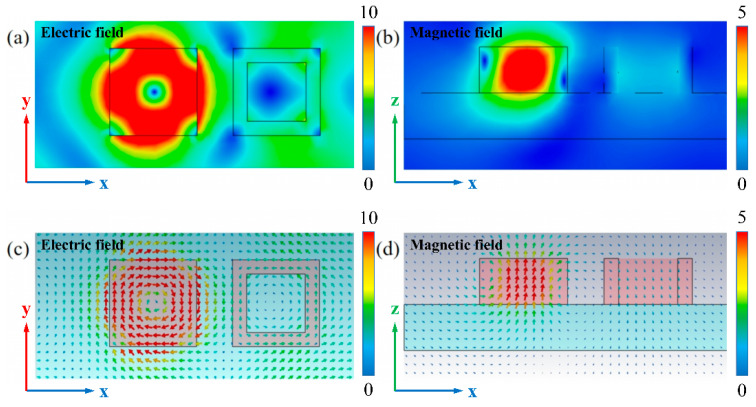
Simulated electric and magnetic field distributions and vector diagrams at the peak position. (**a**,**c**) Top and cross section views of the electric field distribution (x-y plane at z = 95 nm). (**b**,**d**) Top and cross section views of the magnetic field distribution (x-z plane at y = 0).

The electromagnetic field distribution at the transmission peak is further plotted to clarify the EIT generation mechanism in Figure 4. At the resonant position of 1440.4 nm, the electric field in the x-y plane (at z = 95 nm) is mainly distributed on the four sides of the SNC, forming the clockwise rotated electric field. Correspondingly, the clockwise electric field causes a strong magnetic field along the z-axis inside the SNC. Therefore, magnetic resonance occurs in the SNC at the EIT window [32,33,34]. On the other hand, the electric field in the bright HNC is much weaker than that in the SNC. We speculate that bright and dark modes couple at the EIT peak, which results in stronger electric and magnetic field in the SNC. These results also indicate that the dielectric metamaterial can be designed to confine the light field inside the device, which promotes the Si-based nano-scale light–matter interaction and optoelectronic integration.

**Figure 5:**
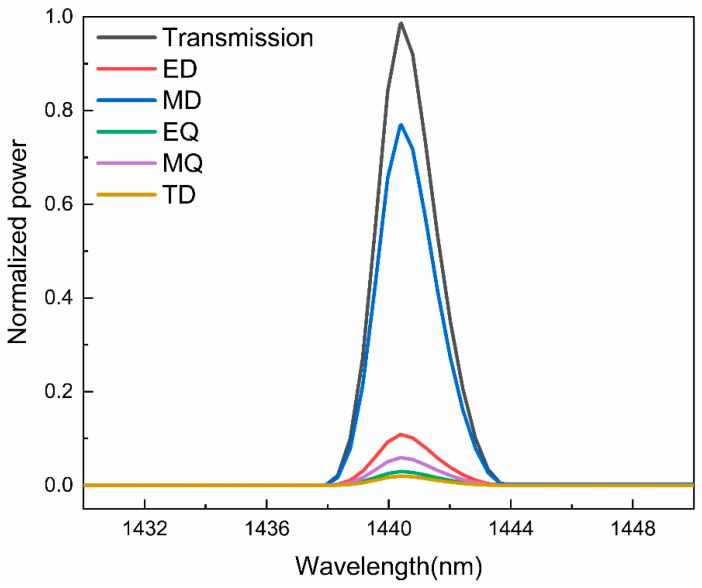
Normalized scattered power of multipoles of the proposed metamaterial, including electric dipole (ED), magnetic dipole (MD), electric quadrupole (EQ), magnetic quadrupole (MQ), and toroidal dipole (TD).

Furthermore, the far-field scattered power in the Cartesian coordinate system is also calculated. Here, we only consider the electric dipole (ED), magnetic dipole (MD), electric quadrupole (EQ), magnetic quadrupole (MQ), and toroidal dipole (TD). The electromagnetic multipole can be expressed as [35]:

(2) $$P=\frac{1}{i\omega }\int jd^{3}r$$

(3) $$M=\frac{1}{2c}\int \left(r\times j\right)d^{3}r$$

(4) $$QE_{\alpha \beta }=\frac{1}{i\omega }\int \left[\left(r_{\alpha }j_{\beta }+r_{\beta }j_{\alpha }\right)-\frac{2}{3}\left(r\times j\right)\right]d^{3}r\;$$

(5) $$QM_{\alpha \beta }=\frac{1}{3c}\int \left[{\left(r\times j\right)}_{\alpha }j_{\beta }+{\left(r\times j\right)}_{\beta }j_{\alpha }\right]d^{3}r$$

(6) $$T=\frac{1}{10c}\int \left[\left(r\cdot j\right)r-r^{2}j\right]d^{3}r$$

where *c* is the speed of light, j is the current density, ω is the angular frequency of electromagnetic wave, and r is the distance vector from the origin to point (x, y, z) in a Cartesian coordinate system. The corresponding far-field scattered power can be expressed as: $I_{P}=\frac{2\omega^{4}}{3c^{3}}{\left|P\right|}^{2}$, $I_{M}=\frac{2\omega^{4}}{3c^{3}}{\left|M\right|}^{2}$, $I_{QE}=\frac{\omega^{6}}{5c^{5}}\sum {\left|QE_{\alpha \beta }\right|}^{2}$, $I_{QM}=\frac{\omega^{6}}{20c^{5}}\sum {\left|QM_{\alpha \beta }\right|}^{2}$, $I_{T}=\frac{2\omega^{6}}{3c^{5}}{\left|T\right|}^{2}$. Based on this, we calculate the normalized scattering power near the EIT peak, as shown in Figure 5. It can be found that the power of MD at the resonance position of EIT is much greater than that of other multipoles. Therefore, the EIT generated in the proposed metamaterial is mainly due to the magnetic dipole resonance.

### 3.2. Dynamic Modulation of the EIT Effect of Graphene-Dielectric Hybrid Metamaterials

**Figure 6:**
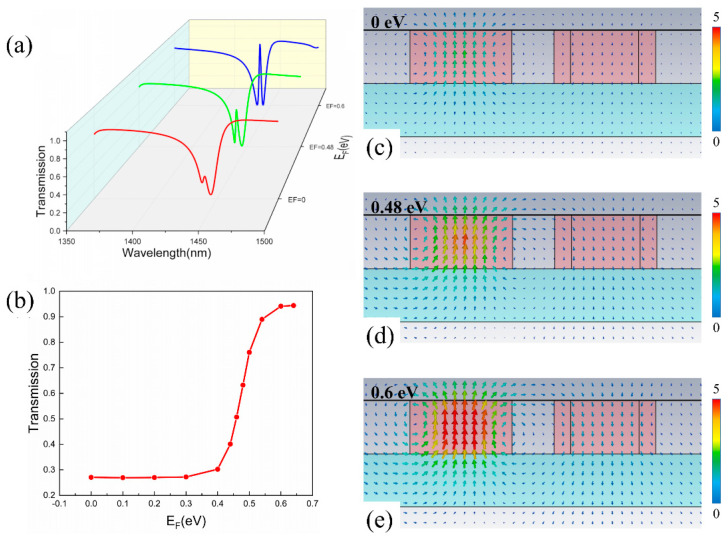
(**a**) The simulated transmission spectrum of the metamaterial when the Fermi level is 0 eV, 0.48 eV, and 0.6 eV, respectively. (**b**) The simulated transmission intensity of an EIT-like peak when the Fermi level changes from 0 eV to 0.7 eV. Simulated magnetic-field distribution in the x-z plane at y = 0 with different Fermi levels. (**c**) 0 eV (**d**) 0.48 eV (**e**) 0.6 eV.

Excellent EIT performance is achieved in the SNC-HNC metamaterial. On this basis, a monolayer graphene is further applied on top to realize the tunability. By changing the Fermi level of graphene, its conductivity changes correspondingly, which further influences its electromagnetic properties and EIT response. As shown in Figure 6a, the EIT peak, which is located at 1439 nm, can be adjusted with different Fermi levels of graphene. With a Fermi level of 0 eV, the spectrum presents a small transmission peak at 1439 nm and the amplitude is less than 0.3. When the Fermi level is above 0.48 eV, the EIT peak increases drastically and reaches a maximum amplitude value of 0.94 at 0.6 eV.

**Figure 7:**
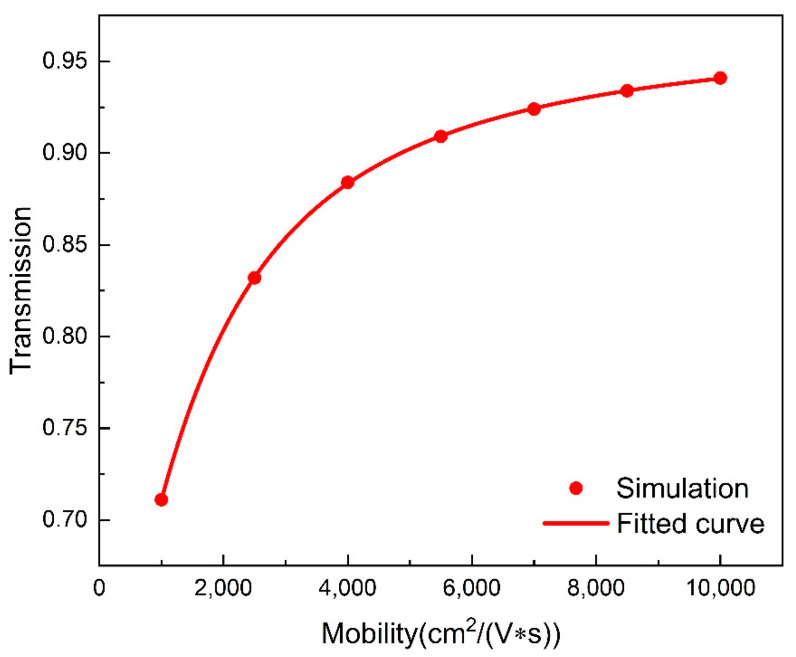
The transmission amplitude of the EIT peak varies with the mobility of graphene carriers (graphene Fermi level is 0.6 eV).

A detailed study is carried out to explore the relationship between the EIT peak amplitude and the Fermi level of graphene. As can be clearly observed in Figure 6b, the transmission peak intensity remains almost unchanged when the Fermi level of graphene changes from 0 eV to 0.4 eV. With a Fermi level greater than 0.4 eV, the peak amplitude increases drastically until reaches the maximum transmittance of 0.9 at 0.6 eV, and the total modulation depth is up to ~70%, which is defined as $\left(T_{peak,max}-T_{peak,min}\right)\times 100\%$. The magnetic field distributions are also investigated as shown in Figure 6c–e. The weakest magnetic field emerges at 0 eV and is strengthened as the Fermi level increases, which is consistent with the change of the EIT peak intensity. Compared with the proposed metamaterial without graphene, the transmission at the EIT position is lower than 0.3 at $E_{F}$ = 0 eV, and the magnetic dipole resonance almost disappears. In this case, the cross-sectional magnetic field of the magnetic dipole resonance strongly couples with the inter-band transition of graphene, resulting in the degradation of transmission and magnetic field. When the Fermi level is above 0.48 eV, the EIT peak increases drastically and reaches a maximum amplitude value of 0.94 at 0.6 eV. Meanwhile, the magnetic field strength is increases gradually. When the Fermi level is greater than half of the photon energy ($E_{F}$ > *ℏω*/2), the contribution of the inter-band transition is prevented due to the Pauli exclusion principle, and the inter-band absorption of graphene declines. Therefore, a fast switch between the high and low EIT peak value is easily attained when the Fermi level changes from 0.4 eV to 0.6 eV, which is especially beneficial to the optical switching applications and ultrasensitive optical devices, etc. Since the carrier mobility of graphene will be different with different fabrication procedures, it is very important to explore the influence of carrier mobility on the EIT performance of the metamaterial. Here, we set the carrier mobility changing from 1000 ${\mathrm{cm}}^{2}/\left(V\cdot s\right)$ to 10,000 ${\mathrm{cm}}^{2}/\left(V\cdot s\right)$. As shown in Figure 7, when the Fermi level of graphene is 0.6 eV, the EIT peak intensity fits logarithmically with the change of carrier mobility. Correspondingly, the graphene-induced modulation shows a similar changing trend.

**Figure 8:**
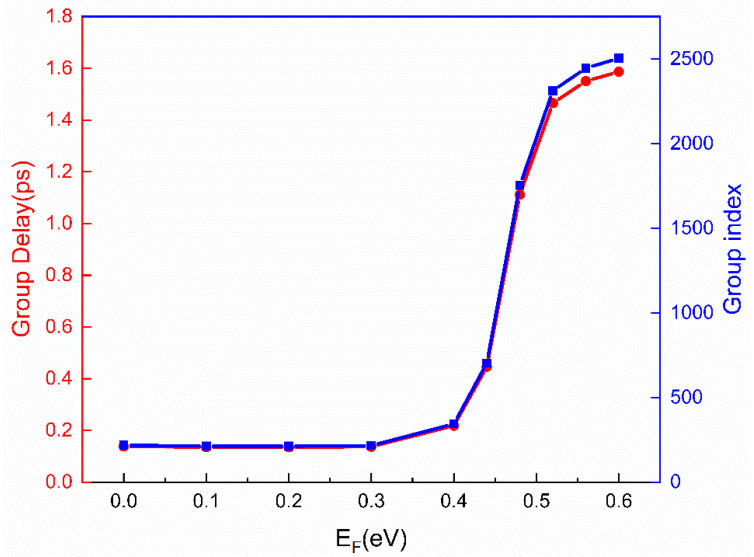
The slow light effect of the proposed metamaterial. Group delay (red) and group index (blue) at different Fermi levels.

The intriguing part of the active EIT effect is its tunable slow light characteristics. EIT-induced strong dispersion emerges and results in the slow group velocity of light. The slow light effect is carefully investigated and the group delay $\tau_{g}$ and group index $n_{g}$ are used to estimate the effect, which can be expressed by the following formula [36]:

(7) $$\tau_{g}=-\frac{d\varphi \left(\omega \right)}{d\omega }$$

(8) $$n_{g}=\frac{c}{v_{g}}=\frac{c}{h}\times \tau_{g}=-\frac{c}{h}\times \frac{d\varphi \left(\omega \right)}{d\omega }$$

where φ is the phase; ω is the frequency; *c* is the speed of light in free space; $v_{g}$ is the group velocity of light; *h* is the thickness of metamaterial structure. The slow light effect is illustrated by the calculated group delay and group index with different Fermi levels of graphene, as shown in Figure 8. When E_F_ is smaller than 0.4 eV, the group delay is about 0.14 ps and the group index is about 220. When the Fermi level is greater than 0.4 eV, both the group delay and group index increase intensively with the increasing Fermi levels, which is consistent with the transmission performance. A high transmittance is obtained when the Fermi level is 0.6 eV and the group delay and group index are as high as 1.6 ps and 2500, respectively. Reasonably, the change of the group delay and group index is basically the same as that of the EIT transmission intensity shown in Figure 6b. Therefore, the tunable slow light effect is ascribed to the graphene covered on top. By changing the Fermi level of graphene between 0.4 eV and 0.6 eV, the group velocity can be flexibly manipulated between c/220 and c/2500 at the transmission peak, which has great application potential in tunable slow light devices.

### 3.3. Refractive Index Sensing

**Figure 9:**
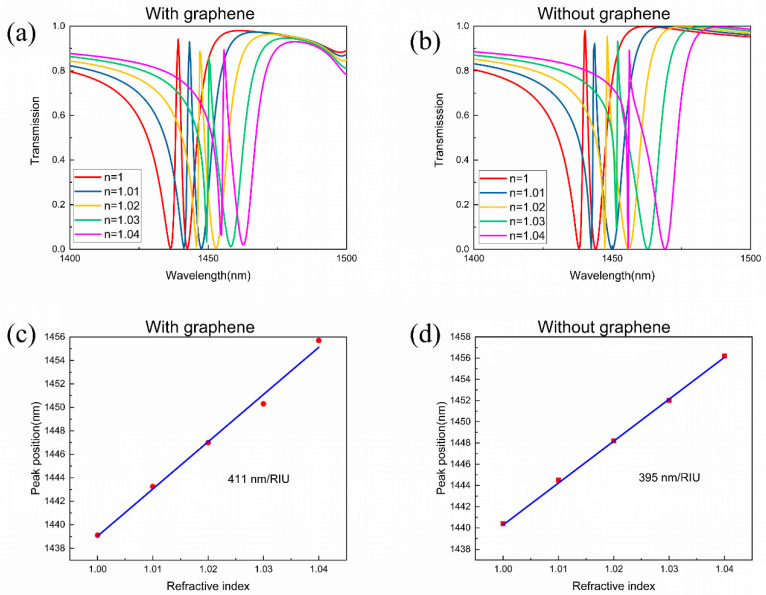
The sensing performance of refractive index. (**a**,**b**) Simulated transmission spectra of the metamaterial with and without graphene when the environmental refractive index changing from 1 to 1.04. (**c**,**d**) The red point is the simulated relationship between the peak position and the background refractive index. The blue curve is a liner fit to the simulated data.

The high refractive index sensitivity is another exhilarating aspect of the EIT effect due to the narrow line width of the transmission peak. When the environmental refractive index varies, the transmission peak shifts sensitively. As shown in Figure 9a, when the refractive index of the surrounding increases, the transmission peak of the proposed metamaterial undergoes a clear redshift and the line width broadens too. The figure of merit (FOM) is an important parameter to evaluate the sensor performance, which is determined by [37]:

(9) $$FOM=\frac{S}{\Delta \lambda },S=\frac{\delta \lambda }{\delta n}$$

where *S* is the shift in the resonance per refractive-index-unit change; Δ*λ* is the line width of the transmission peak. Figure 9c shows the trend of the peak position changes with the refractive index in an approximately liner relationship, and its slope represents the refractive index sensitivity of the metamaterial. By calculation, the refractive index sensitivity of the graphene-covered metamaterial is *S* = 411 nm/RIU, which is better than the reported Si metamaterial [38]. Combined with the average line-width Δ*λ* = 2.58 nm, the FOM is calculated as 159, which is higher than previously reported results in the near-infrared region [13]. Next, in order to explore the influence of graphene on the refractive index sensing, we compared the sensing performance of the proposed metamaterial with and without graphene. It can be seen from Figure 9a,b that the EIT peak undergoes an apparent red-shift with the increase of the environmental refractive index, regardless of whether there is graphene or not. However, in the condition without graphene, the line width of the EIT peak is greatly increased during the red shift, and the peak shape is severely deformed. Moreover, the sensor performance of the metamaterial without graphene is lower than the graphene-covered one, in which the refractive index sensitivity is 395 nm/RIU and the FOM is 106. The difference can be explained in this way: The incident electromagnetic wave excites the surface plasmon polaritons in graphene, which further enhance the light absorption and the mode coupling in the metamaterial. Therefore, the graphene-covered metamaterial can effectively improve the refractive index sensitivity and suppress the deformation of the EIT peak caused by the change of the environmental refractive index.

## 4. Conclusions

In conclusion, a graphene-dielectric hybrid metamaterial with active manipulation of the EIT effect in the near-infrared region is proposed. Due to the magnetic resonance of the SNC, a characteristic EIT transmission peak with a high Q-factor of 646 and high transmission of 97.5% is observed. By changing the Fermi level of graphene, dynamic control of the EIT peak is observed and the modulation depth can reach up to 70% with a sharp switch. Originating from the EIT performance, the intriguing slow light effect is attained, and the group velocity can be continually tuned from c/200 to c/2500. Moreover, a high sensitivity of the refractive index is also realized with a FOM value up to 159, which is higher than the previously reported results in the near-infrared region. Compared to the metamaterial without graphene, the sensing performance is significantly improved. Therefore, the proposed metamaterial presents excellent EIT performance with a tunable transmission peak, a changeable group velocity, and high refractive index sensitivity, which highlights its applications in optical switching, tunable slow light devices, and high-sensitivity sensors, etc. Moreover, the accessible all-dielectric structure based on graphene and Si will greatly promote the nanoscale light–matter interaction and silicon-based optoelectronic integration.
